# Novel Flow Cytometry Analyses of Boar Sperm Viability: Can the Addition of Whole Sperm-Rich Fraction Seminal Plasma to Frozen-Thawed Boar Sperm Affect It?

**DOI:** 10.1371/journal.pone.0160988

**Published:** 2016-08-16

**Authors:** Mariana Andrade Torres, Rommy Díaz, Rodrigo Boguen, Simone Maria Massami Kitamura Martins, Gisele Mouro Ravagnani, Diego Feitosa Leal, Melissa de Lima Oliveira, Bruno Bracco Donatelli Muro, Beatriz Martins Parra, Flávio Vieira Meirelles, Frederico Ozanan Papa, José Antônio Dell’Aqua, Marco Antônio Alvarenga, Aníbal de Sant’Anna Moretti, Néstor Sepúlveda, André Furugen Cesar de Andrade

**Affiliations:** 1 Laboratory of Andrology and Technology of Swine Embryos, Department of Animal Reproduction, School of Veterinary Medicine and Animal Science, University of São Paulo, Pirassununga, São Paulo, Brazil; 2 Center of Excellence in Biotechnology of Reproduction, University of La Frontera, Temuco, Araucania, Chile; 3 Laboratory of Swine Research, Department of Animal Nutrition and Production, School of Veterinary Medicine and Animal Science, University of São Paulo, Pirassununga, São Paulo, Brazil; 4 Department of Veterinary Medicine, School of Animal Sciences and Food Engineerig, University of São Paulo, Pirassununga, São Paulo, Brazil; 5 Department of Animal Reproduction and Veterinary Radiology, School of Veterinary Medicine and Animal Science, São Paulo State University (UNESP), Botucatu, São Paulo, Brazil; University Hospital of Münster, GERMANY

## Abstract

Boar semen cryopreservation remains a challenge due to the extension of cold shock damage. Thus, many alternatives have emerged to improve the quality of frozen-thawed boar sperm. Although the use of seminal plasma arising from boar sperm-rich fraction (SP-SRF) has shown good efficacy; however, the majority of actual sperm evaluation techniques include a single or dual sperm parameter analysis, which overrates the real sperm viability. Within this context, this work was performed to introduce a sperm flow cytometry fourfold stain technique for simultaneous evaluation of plasma and acrosomal membrane integrity and mitochondrial membrane potential. We then used the sperm flow cytometry fourfold stain technique to study the effect of SP-SRF on frozen-thawed boar sperm and further evaluated the effect of this treatment on sperm movement, tyrosine phosphorylation and fertility rate (FR). The sperm fourfold stain technique is accurate (R^2^ = 0.9356, p > 0.01) for simultaneous evaluation of plasma and acrosomal membrane integrity and mitochondrial membrane potential (IPIAH cells). Centrifugation pre-cryopreservation was not deleterious (p > 0.05) for any analyzed variables. Addition of SP-SRF after cryopreservation was able to improve total and progressive motility (p < 0.05) when boar semen was cryopreserved without SP-SRF; however, it was not able to decrease tyrosine phosphorylation (p > 0.05) or improve IPIAH cells (p > 0.05). FR was not (p > 0.05) statistically increased by the addition of seminal plasma, though females inseminated with frozen-thawed boar semen plus SP-SRF did perform better than those inseminated with sperm lacking seminal plasma. Thus, we conclude that sperm fourfold stain can be used to simultaneously evaluate plasma and acrosomal membrane integrity and mitochondrial membrane potential, and the addition of SP-SRF at thawed boar semen cryopreserved in absence of SP-SRF improve its total and progressive motility.

## Introduction

Due to reductions in fertility and litter size rates, frozen-thawed (FT) boar semen has only been commercially used on a small scale [1,2]. This decreased rates occur because cryopreservation leads to structural injuries in the sperm plasma and acrosomal membranes and causes functional damage such as capacitation-like changes, which decrease fertilizing potential [3].

Seminal plasma (SP) has been studied as a method of minimizing/reversing cryopreservation injuries. In addition, ejaculate-specific fractions from boars can be used as an alternative to whole ejaculate [4]. Utilization of a sperm peak portion (first 10 ml of the sperm-rich fraction—SRF) has also shown promising results for improving boar cryopreservation [5–8]. However, we believe that sperm cryopreservation can achieve better results with the use of the whole SRF, which corresponds to both the sperm-peak portion and the remaining part of the SRF. The benefits may be because the sperm-peak portion has a sperm concentration of approximately 16 x 10^9^ cells of the total ejaculated fraction and the remaining part of the SRF approximately 15 x 10^9^ cells [9]. However, the use of only the sperm-peak portion has an associated loss of spermatozoa that cannot be compensated for by its benefits.

Although the addition of frozen-thawed boar SP results in an improvement in motility as well as membrane integrity [10,11], improvements in fertility have scarcely been attained [12]. This may be because sperm evaluations overestimate good spermatozoa when only individual characteristics are analyzed. It is worth mentioning that, there is no evaluation of sperm function that can assess real sperm fertility, which depends not only on the spermatozoa but also on multiple factors [13]. Despite the difficulty in developing an accurate single laboratory test for predicting a semen sample’s fertility potential [14,15], evaluation of multiple sperm function parameters in a given sample, compared to analyses of individual characteristics, could estimate fertilizing potential with greater chances of success [16].

An epifluorescence microscopy technique previous described by Andrade et al. [17] and Celeghini et al. [18] allows simultaneous evaluation of three essential sperm functions: integrity of the plasma membrane, integrity of the acrosomal membrane and mitochondrial membrane potential (Δψm) [17,18]. This technique is accomplished by combining the following probes: Hoechst 33342, a DNA stain able to differentiate cell debris and spermatozoa; Propidium Iodide (PI), a DNA stain that indicates plasma membrane damage; FITC conjugated with *Pisium Sativum agglutinin* (PSA), which binds to α-D- glucosyl and α-D-mannosyl residues of a damaged acrosome [19]; and 5,5’,6,6’-tetrachloro-1,1’,3,3’-tetraethylbenzimidazolyl carbocyanine iodide (JC-1) for evaluating Δψm. This technique allows to classify the spermatozoa by up to eight subpopulations [17,18].

However, there is a limit to the number of spermatozoa counted by epifluorescence microscopy technique. Indeed, a larger amount of cells can be analyzed in a short time period using flow cytometry, increasing the accuracy of such technique [20,21]. Nevertheless, to date, it has not been possible to use this probe combination with flow cytometry. Currently, sperm flow cytometry analysis protocols for assaying sperm viability are generally used with double stain, for individual sperm parameter evaluation, as the association of PI, (an impermeable plasma membrane probe) with SYBR-14 [14,22,23] or other DNA stains with low plasma membrane selectivity [24–26]. Another sperm staining protocol that is commonly used is the triple stain method, which evaluates plasma membrane integrity with PI and acrosomal status with lecithin conjugated to a fluorophore such as PSA (*Pisum sativum* agglutinin) or PNA (*Arachis hypogaea* agglutinin) or other glycoprotein markers. A DNA stain for excluding cell debris that is the same size as spermatozoa can be include in the staining protocol [14,27]. Mitochondrial membrane potential (Δψm) had been assessed using flow cytometry, which can be accomplished with JC-1, Mitotraker green FM or Rhodamine 123 in association with PI and a DNA stain [28,29]. Furthermore, a membrane-permeating fluorescent probe is needed for flow cytometry sperm analysis, and its absence affects the ability to distinguish spermatozoa from cellular debris and particles with similar light scattering [13,30].

Therefore, the aim of this study was to (1) introduce a flow cytometry fourfold stain technique for simultaneously evaluating of plasma, acrosome and mitochondrial membrane excluding cell debris; (2) evaluate these parameters in association with sperm kinetics, tyrosine phosphorylation and *in vivo* fertility in FT boar semen containing 10% of seminal plasma arising from whole sperm-rich fraction (SP-SRF) in an attempt to confirm the role of this on sperm physiology and fertility and (3) whether centrifugation has an effect on the post-thawed boar semen quality, enabling separation of the centrifugation and the SP-SRF effects.

## Material and Methods

### Reagents and chemicals

The freezing medium (Botu-Sui^®^ - composed of sugars, amino acids, buffers, 20% egg yolk [v/v], antibiotics, 2% glycerol as a cryoprotectant, and 2% methylformamide [v/v]) was developed and donated by Biotech-Botucatu- Ltda ⁄ME (Botucatu, SP, Brazil). Beltsville Thawing Solution (BTS) was acquired from IMV Technologies (L'Aigle, France). The hormones used were purchased from MSD Animal Health (New York, EUA—Regumate^®^), Bioneche Animal Health (Ontario, Canada—Lutropin^®^- V) and Coopers (Brazil—Novormon^®^). Fetal bovine serum was purchased from Vitrocell—Embriolife (Campinas, Brazil). Hoechst 33342 and JC-1 fluorescent probes were purchased from Molecular Probes (Eugene, OR, USA). Unless otherwise stated, all other chemicals (propidium iodide [PI], *Pisum sativum* agglutinin conjugated to FITC [PSA] and an anti-phosphotyrosine antibody conjugated to fluorescein) were purchased from Sigma-Aldrich (St. Louis, MO, USA).

### Experiment 1: Flow cytometry application in the method with fourfold stain for simultaneous assessment of plasma, acrosomal and mitochondrial membranes of boar sperm

#### Staining incubation medium

Semen samples were diluted in Tyrode’s albumin lactate pyruvate (TALP) sperm medium [31]. The medium pH was adjusted to 7.4 using 5 M NaOH.

#### Flow cytometry analysis

Flow cytometry was performed with a BD FACSAria flow cytometer (Becton Dickinson, San Jose, CA, USA) controlled by BD FACSDiva 6.0 software (Becton Dickinson). An argon laser at 488 nm and a near-UV laser at 375 nm simultaneously excited the cells. Quality control of the FACSAria operation was performed before every routine analysis using CST Software (Cytometer Setup and Tracking). When necessary, manual compensation of the sperm flow cytometry analysis was performed with the aid of a positive control for each probe used (S1 Fig). The samples were processed at an acquisition rate of approximately 600–1000 events/s, acquiring 10,000 cells per analysis. To exclude particles without nuclei and with the same scatter properties as spermatozoa, samples of 150 μL diluted in TALP medium to 5 x 10^6^ spermatozoa/mL were stained with Hoechst 33342 (H342, 2.33 μg/mL) for 10 minutes at 37°C [32]. The following detectors were used for the analysis: H342—band pass 450/40 nm; PI—long pass 655 nm and band pass 695/40; PSA—band pass 530/30 nm; and JC-1 (5,5’,6,6’-tetra-chloro-1,1’,3,3’-tetraethylbenzimidazolyl carbocyanine iodide)—long pass 556 nm and band pass 585/42 nm.

#### Semen collection and raw evaluation

A single sperm-rich fraction was collected from nine boars (n = 9) using the gloved-hand technique. Immediately after collection, the semen samples were diluted in TALP sperm medium pre-warmed to 37°C and adjusted to a final concentration of 25 x 10^6^ spermatozoa/mL using a Neubauer hemocytometer. The semen was evaluated for motility characteristics using a sperm class analyzer (SCA—Microptics^®^ - Barcelona/Spain).

#### Sample preparation

Raw semen was diluted in TALP medium to a final concentration of 5 x 10^6^ spermatozoa/mL and split into two aliquots: one was kept its raw diluted conditions, and the other was subjected to three cycles of flash freezing in liquid nitrogen and slow thawing to induce damage to the acrosomal and plasma membranes and to perturb mitochondrial function. Five treatments were prepared with the following fixed ratios of raw diluted semen to flash frozen semen: 100: 0 (T100), 75: 25 (T75), 50: 50 (T50), 25: 75 (T25) and 0: 100 (T0). After treatment allocation, total motility was evaluated using an SCA (Microptics^®^ - Barcelona/Spain).

#### Sperm flow cytometry fourfold stain

**Fig 1:**
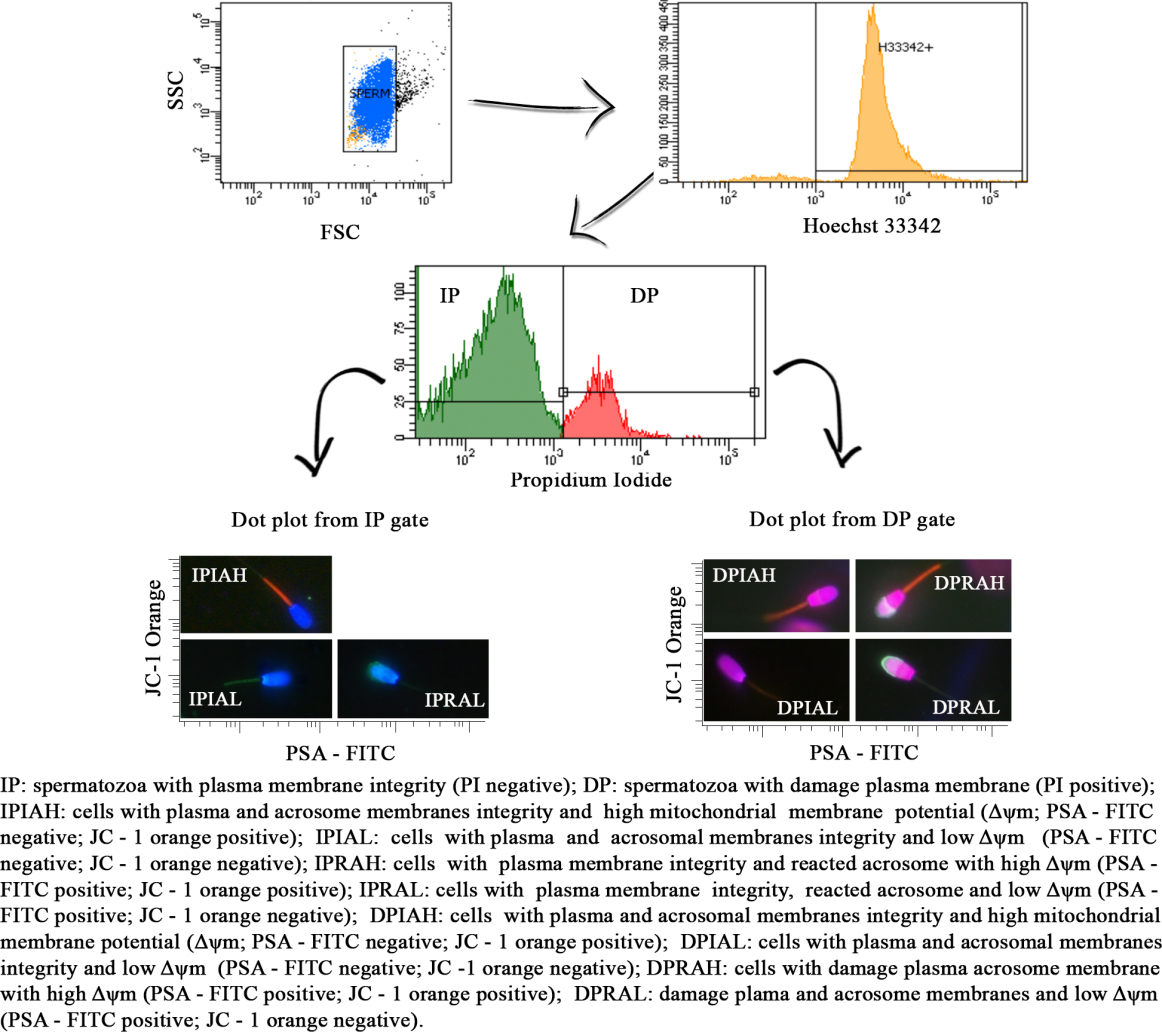
Analysis of flow cytometry fourfold stain for simultaneous assessment of plasma, acrosomal and mitochondrial membranes of boar sperm: an epifluorescence view. (A) Side scatter (SSC) x forward scatter (FSC) dot plot showing the “Sperm + cell debris” gate. (B) Hoechst 33342 histogram originating from the “Sperm + cell debris” gate to exclude non-cellular particles by a DNA probe, Hoechst 33342. (C) Propidium iodide histogram based on Hoechst 33342 for evaluating sperm cell membrane integrity. IM gate showing spermatozoa with intact plasma membranes and the DM gate showing damaged sperm plasma membranes. (D) Dot plot from the IM gate for analyzing mitochondrial membrane potential (axis y; JC-1 orange) and acrosome integrity (axis x; PSA-FITC), as represented by each quadrant of expected sperm cells. (E) Dot plot from the DM gate for analyzing mitochondrial membrane potential (axis y; JC-1 orange) and acrosome integrity (axis x; PSA-FITC), as represented by each quadrant of expected sperm cells.

The sperm flow cytometry fourfold stain method aimed to incorporate the flow cytometry in the technique previous described for the same staining approach using epifluorescence microscopy [17,18], allowing a technique of rapid and accurate sperm analysis (Fig 1). This technique allows the detection of eight sperm populations named: IPIAH–simultaneous plasma and acrosome membrane integrity and high mitochondrial membrane potential (Δψm); IPIAL—simultaneous plasma and acrosome acrosomal integrity and low Δψm, IPRAH—simultaneous plasma membrane integrity, reacted acrosome and high Δψm, IPRAL—simultaneous plasma membrane integrity, reacted acrosome and low Δψm, DPIAH—simultaneous damaged plasma membrane, acrosome integrity and high Δψm, DPIAL—simultaneous damaged plasma membrane, acrosome integrity and low Δψm, DPRAH—simultaneous damaged plasma membrane, reacted acrosome and high Δψm, DPRAL—simultaneous damaged plasma membrane, reacted acrosome and low Δψm. The sperm population IPIAH could be named as: potentially fertile spermatozoa; since it presents simultaneous plasma and acrosomal membranes integrity, and high mitochondrial potential, which are essential characteristics to sperm fertility.

Each treatment aliquot (150 μL with 5 x 10^6^ spermatozoa/mL) was stained with H342 (2.33 μg/ mL), PI (100 μg/ mL), PSA (13.3 μg/ mL) and JC-1 (4.08 μM). Samples were incubated at 37°C for 10 min, diluted in 150 μL TALP medium and analyzed by flow cytometry.

#### Sperm flow cytometry dual and triple stain

Same samples (single ejaculate collected by gloved-hand technique from 9 cross-bread boars) used to sperm flow cytometer fourfold stain was used to dual and triple stain assays. For dual stain analysis (individual sperm compartment was evaluated–Table 1), each treatment aliquot (150 μL with 5 x 10^6^ spermatozoa/mL) was stained with H342 associated with: PI or PSA or JC-1. To perform triple stain (two sperm compartments evaluated–plasma and acrosome membranes–Table 1), each treatment aliquot was stained with H342 associated with PI and PSA. Dual and triple stain analysis was performed with the treatments previous described for fourfold stain, which were performed with the following fixed ratios of raw diluted semen to flash frozen semen: 100: 0 (T100), 75: 25 (T75), 50: 50 (T50), 25: 75 (T25) and 0: 100 (T0).

**Table 1:** Differentiation of stain type regards sperm compartment evaluated.

Stain type	Sperm Compartment Evaluated			Exclusion of Cell debris	Cell category
	Plasma Membrane	Acrosome	Mitochondrial Potential		
Dual stain^1^	+	−	−	+	IP
	−	+	−	+	IA
	−	−	+	+	HP
Triple stain^2^	+	+	−	+	IPIA
					IPRA
					DPIA
					DPRA
Fourfould stain^3^	+	+	+	+	IPIAH
					IPIAL
					IPRAH
					IPRAL
					DPIAH
					DPIAL
					DPRAH
					DPRAL

IP–integrity of plasma membrane, IA–integrity of acrosome, HP–high mitochondrial membrane potential (Δψm), IPIA–simultaneous integrity of plasma and acrosome membranes, IPRA–simultaneous integrity of plasma membrane and reacted acrosome, DPIA–simultaneous damage of plasma membrane and integrity of acrosome, DPRA–simultaneous damage of plasma and acrosome membranes, IPIAH–simultaneous plasma and acrosome membrane integrity and high Δψm, IPIAL—simultaneous plasma and acrosome acrosomal integrity and low Δψm, IPRAH—simultaneous plasma membrane integrity, reacted acrosome and high Δψm, IPRAL—simultaneous plasma membrane integrity, reacted acrosome and low Δψm, DPIAH—simultaneous damaged plasma membrane, acrosome integrity and high Δψm, DPIAL—simultaneous damaged plasma membrane, acrosome integrity and low Δψm, DPRAH—simultaneous damaged plasma membrane, reacted acrosome and high Δψm, DPRAL—simultaneous damaged plasma membrane, reacted acrosome and low Δψm.

^1^Dual stain was performed by the association of Hoechst 33342 with propidium iodide (PI for IP analysis), or *Pisum sativum* agglutinin conjugated to FITC (PSA for IA analysis), or JC-1 fluorescent probes (for HP analysis)

^2^Triple stain was performed by the association of Hoechst 33342 with PI and PSA

^3^Fourfold stain was performed by the association of Hoechst 33342 with PI, PSA and JC-1.

### Experiment 2: *In vitro* assay: effect on boar spermatozoa quality of the addition of whole sperm-rich fraction seminal plasma to frozen-thawed semen

#### Semen collection, raw semen evaluation and freezing

Four whole sperm-rich fractions were obtained from each of six boars (n = 24). Immediately after collection, the concentration was evaluated, and CASA analyses were performed. After the initial analysis, the semen was distributed into three treatments: a control (CT–without handling of seminal plasma); suspended in autologous seminal plasma after centrifugation (CS) and withdrawn seminal plasma after centrifugation (CW). The CS sediment was suspended in its own seminal plasma after centrifugation (500 x g/10 min). The CW samples were centrifuged (500 x g/10 min), and the supernatant was completely separated from the pellet by aspiration, reserved and processed as described below in “Seminal plasma collection and storage”. The CT, CS and pellet fractions obtained from CW were suspended at ambient temperature (25°C) using a freezing extender containing 2% glycerol and 2% methylformamide [v/v], as a cryoprotectants, to obtain a final concentration of 300 x 10^6^ spermatozoa/mL and stored in 0.5 mL straws (IMV, Laigle, France). The straws were subsequently placed into an automatic freezing system (TK 3000^®^; TK Tecnologia em Congelação Ltda, Uberaba, Brazil) and cooled at a rate was -0.5°C⁄ min from 25°C to 5°C. The freezing rate was -20°C⁄ min from 5 to -120°C. Subsequently, the straws were immersed in liquid nitrogen at -196°C and stored in goblets within cryogenic tanks. All straws were kept in liquid nitrogen for a minimum of one week before thawing.

#### Seminal plasma arising from sperm-rich fraction: collection and storage

Autologous seminal plasma arising from sperm-rich fraction (SP-SRF) was obtained from the same sample collection for cryopreservation. The supernatant previously obtained was centrifuged (2500 x g/ 30 min) and completely separated from the pellet by aspiration. This sample was then vacuum filtered through disposable filters (0.22 μm in diameter; 99150—Filtermax, TPP^®^; Switzerland) and stored at -80°C for further use.

#### Semen thawing and distribution into a novel treatment

Two straws per ejaculate and treatment (CT, CS and CW) were thawed in a water bath at 37°C for 30 sec and diluted to a final concentration of 25 x 10^6^ spermatozoa/mL in a freezing extender. Additionally, two straws per ejaculate from the CW treatment were thawed and diluted with freezing extender supplemented with 10% SP-SRF (v:v) [33] to generate the treatment CWSP (CW containing 10% of SP-SRF). After allocation into treatments, the samples were kept in a water bath at 37°C until all analyses were concluded. An aliquot was withdrawn from each experimental group at three time points, 0 (allowed to equilibrate for 5 min), 60, and 120 min, and analyzed after each staining protocol.

#### Computer-Assisted Sperm Analysis (CASA)

A sample aliquot (5 μL) was withdrawn at each incubation time point and placed on a pre-warmed cover slide and evaluated by phase-contrast microscopy (Nikon, Modelo Eclipse 80i) with 100x magnification. Five good fields were examine using SCA (Microptics^®^ - Barcelona/Spain) for evaluating the following parameters: total (TM) and progressive (PM) motility, curvilinear velocity (VCL), straight-line velocity (VSL), average path velocity (VAP), linearity (LIN), straightness (STR), amplitude of lateral head displacement (ALH), beat cross frequency (BCF) and hypermotility (HIPER). The hyperactivated sperm population was evaluated using Edit/Sort in the software, with ALH > 3.5 μm and VCL > 97 μm/s [34].

#### Sperm flow cytometry fourfold stain

Samples were stained and analyzed as described above in the section “Sperm flow cytometry fourfold stain.”

#### Detect of tyrosine phosphorylation on the sperm surface

Semen samples were analyzed for the presence of protein tyrosine phosphorylation on the surface of the sperm membrane [35]. Samples were diluted in TALP to a final concentration of 2 x 10^6^ sperm/mL in 141 μL. An anti-phosphotyrosine antibody conjugated to fluorescein (F0426, 2 μg/mL) and PI (100 μg/ mL) was added to the H342-stained sample. The sample was incubated for 5 min at 37°C and subjected to flow cytometry [36]. The mean intensity of fluorescence emission (a.u.) captured in a 530/30 nm band pass filter [37] was analyzed for the anti-phosphotyrosine antibody-labeled surface of viable cells (PI negative) [32,38].

### Experiment 3: *In vivo* assay: fertility rate of frozen-thawed boar semen added of whole seminal plasma arising from sperm-rich fraction

#### Semen collection, raw semen evaluation and freezing

Six sperm-rich fractions were obtained as described above, from each of two of the best boars based on the results of experiment 2 to sperm viability. The insemination dose was prepared using the pooled sperm-rich fraction of those two boars. This assay was performed only with the CT, CW and CWSP treatments and was carried out as described above. The exclusion of CS treatment was possible only because any centrifugation effect was observed in experiment 2 (see ‘Results‘ > ‘Experiment 2’). Cryopreservation was also performed using the same protocols described in experiment 2.

#### Seminal plasma arising from sperm-rich fraction: collection and storage

Whole homologous SP-SRF was obtained from the same two boars used for semen cryopreservation. Sperm-rich fractions were collected using the glove-hand technique and evaluated using the CASA system. Samples with total motility ≥ 80% were pooled and centrifuged twice (500 x g/10 min and 2500 x g/ 30 min). The recovered supernatant was vacuum filtered and stored as previously described.

#### Thawing and addition of seminal plasma arising from sperm-rich fraction

An insemination dose was prepared in 50 mL of BTS (Beltsville Thawing Solution) with 30 x 10^6^ spermatozoa/ mL. Ten straws of CT and CW were thawed in a water bath at 37°C for 30 sec, and 45 mL of BTS pre-warmed to 37°C was slowly added to the thawed semen. The other ten straws of CW were thawed under the same conditions, and 40 mL of BTS plus 10% homologous SP-SRF (v:v) was slowly added [33], generating the CWSP treatment.

#### Gilt hormonal protocol and intra-uterine insemination

Thirty-three gilts were fixed-time inseminated (eleven per treatment; n = 33). The females were orally treated with altrenogest (4 mg/ mL; Regumate^®^) for 18 days. Twenty-four hours after altrenogest withdrawal, 600 UI of eCG (equine chorionic gonadotropin; Novormon^®^) was administered [39]. Within a 72 hour interval [40], 2.5 mg of luteinizing hormone (LH; Lutropin^®^) was administered [41]. Only one intra-uterine artificial insemination (IUAI) was performed at 36 hours after ovulation induction [40].

#### Gilt slaughter and embryo collection

Five days after IUAI, the gilts were slaughtered for reproductive tract collection. This procedure complied with the legal standards and ethics of the Ethics Committee on the use of Animals at the School of Veterinary Medicine and Animal Science of University of São Paulo, which approved this study under protocol 3066/2013. Before electro-stunning, a pipette was introduced into the cervix to prevent urine reflux. After bloodletting, the reproductive tract was withdrawn using a linea alba incision. Each ovary was identified, and the corpus luteum was counted to verify the fertility rate. The oviduct and cranial uterine horn were dissected, separated and flushed with 20 mL of PBS (phosphate buffered saline) containing 1% fetal bovine serum. The lavage was collected in a pre-heated Petri dish and evaluated under 20x magnification. The fertility rate (FR–number of embryos/sum of oocytes and embryos) was calculated after embryo collection from the flushed oviducts.

### Statistical analysis

*Experiment 1* consisted of a randomized block design (each ejaculate was considered a block) with five treatments arranged in levels (T0, T25, T50, T75 and T100) and was evaluated by linear regression. The Pearson linear correlation was calculated between IPIAH and total motility, and both were analyzed using SAS MIXED procedure. Sperm categories were analyzed separately using Tukey’s test. *Experiment 2* consisted of a randomized block design with repeated measurement, and each boar was considered a block. For variables that had no interaction between time and treatment, the effects of time (0, 60 and 120 minutes) and treatments (CT, CS, CW and CWSP) on sperm motility, IPIAH cells and tyrosine phosphorylation were analyzed separately using the Tukey-Kramer SAS MIXED procedure. *Experiment 3* consisted of a randomized design. The fertility rate was analyzed using the SAS GLM procedure. All statistical analyses were considered significant at p < 0.05, and all results are expressed as the means ± SEM.

## Results

### Experiment 1: Flow cytometry application in the method with fourfold stain for simultaneous assessment of plasma, acrosomal and mitochondrial membranes of boar sperm

**Fig 2:**
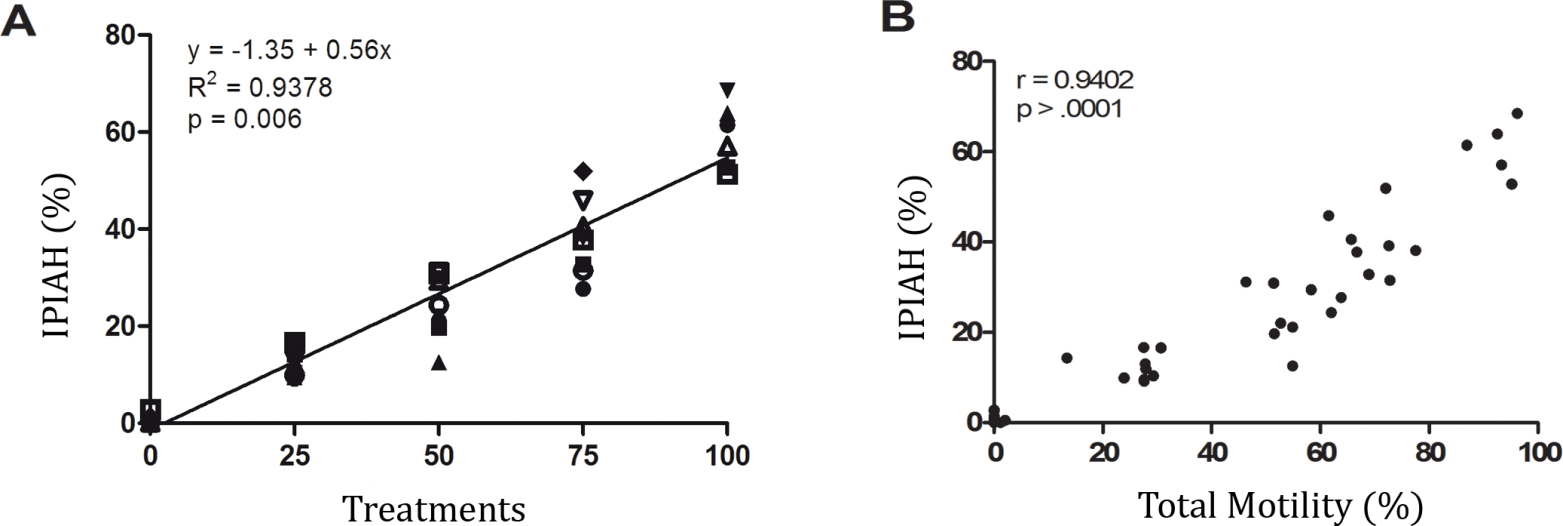
Linear regression and correlation. (A) Linear regression between treatments of the sperm population with plasma and acrosomal membrane integrity and high mitochondrial membrane potential (IPIAH). (B) Correlation between sperm IPIAH and total motility.

**Fig 3:**
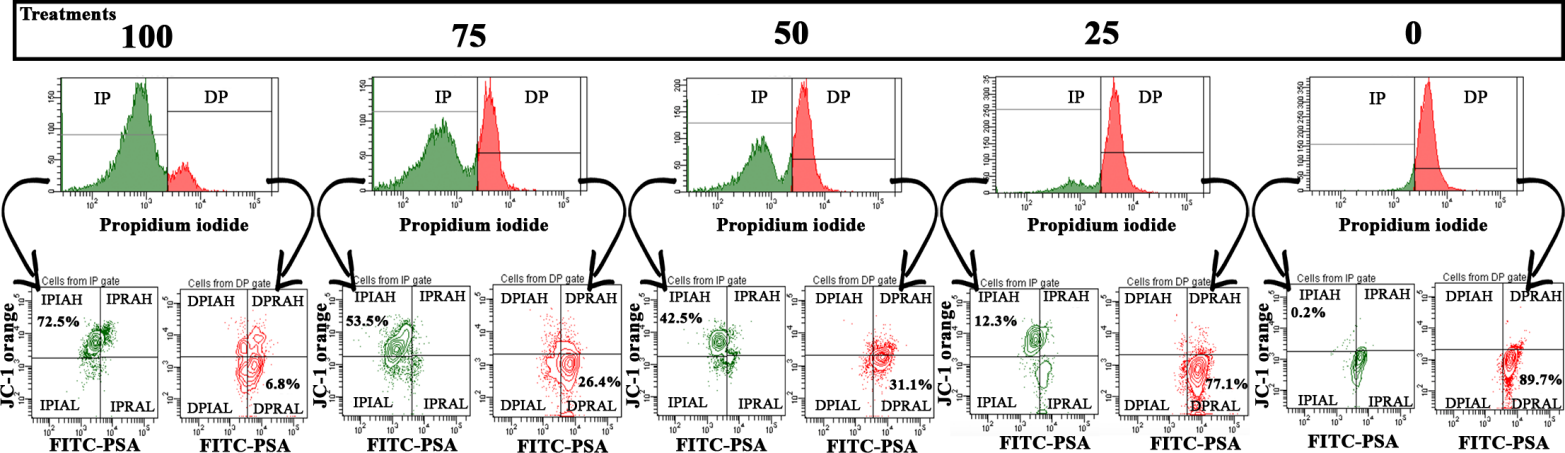
Treatment dynamics. Graphic representation of all semen treatment dynamics analyzed using three graphs: a histogram that represents the plasma membrane integrity and two dot plots for analyzing mitochondrial membrane potential (axis y) and acrosome integrity (axis x). The dot plot with green stain represents intact plasma sperm membranes (from the IP histogram gate–plasma membrane integrity), and the dot plot with red coloration represents damaged plasma sperm membranes (from the DP histogram gate–damaged plasma membranes). IPIAH–simultaneous plasma and acrosome membrane integrity and high mitochondrial membrane potential (Δψm), IPIAL—simultaneous plasma and acrosome acrosomal integrity and low Δψm, IPRAH—simultaneous plasma membrane integrity, reacted acrosome and high Δψm, IPRAL—simultaneous plasma membrane integrity, reacted acrosome and low Δψm, DPIAH—simultaneous damaged plasma membrane, acrosome integrity and high Δψm, DPIAL—simultaneous damaged plasma membrane, acrosome integrity and low Δψm, DPRAH—simultaneous damaged plasma membrane, reacted acrosome and high Δψm, DPRAL—simultaneous damaged plasma membrane, reacted acrosome and low Δψm.

Flow cytometry application of the sperm fourfold stain using combined Hoechst 33342, propidium iodide, lecithin from *Pisum sativum* conjugated to FITC and JC-1 staining allows the detection of eight sperm populations previous explained and named: IPIAH, IPIAL, IPRAH, IPRAL, DPIAH, DPIAL, DPRAH and DPRAL. This technique was effective (R^2^ = 0.9378; p = 0.006) for simultaneously detecting boar sperm with plasma and acrosomal integrity and high mitochondrial membrane potential (IPIAH—Fig 2A). To verify IPIAH sperm population behavior, the Pearson linear correlation was calculated between IPIAH and total motility. As expected, the IPIAH sperm population was highly positively correlated (r = 0.9402; p = >.0001) with total motility (Fig 2B). Fig 3 verifies the treatment dynamics; the sperm population IPIAH declines as the ratio of undamaged semen to flash-frozen semen declines.

Sperm assay by dual or triple stain, in the same sample, overrates spermatozoa potentially fertile. It is noted when raw diluted samples (T100) were evaluated by dual stain, and the samples exhibited 76.07 ± 3.83% of plasma membrane integrity, 95.14 ± 0.64% of acrosome integrity and 76.33 ± 3.14% of high mitochondrial membrane potential (Table 2). On the other way, the simultaneous analysis of plasma and acrosome membranes integrity (triple stain–Table 1) shown 75.24 ± 3.83% of sperm viability (Table 2); and the addition of mitochondrial potential evaluation simultaneous to plasma and acrosome membrane analysis (fourfold stain—Table 1) showed 59.17 ± 2.17% of potentially fertile spermatozoa (Table 2).

**Table 2:** Mean ± standard error of different cell category obtained from different stain type, in bases of percentage (%) of raw diluted semen.

Stain type	Cell category	Percentage (%) of raw diluted semen				
		0	25	50	75	100
Dual stain^1^	IP	18.69 ± 6.04 ^c^	21.63 ± 2.27 ^b, c^	37.60 ± 3.32 ^b, c^	53.49 ± 4.84 ^b^	76.07 ± 3.83^a^
	IA	39.92 ± 8.90 ^c^	50.81 ± 8.14 ^b, c^	62.94 ± 6.10 ^a, b, c^	76.66 ± 4.72 ^a, b^	95.14 ± 0.64 ^a^
	HP	8.14 ± 3.05 ^e^	19.65 ± 1.79 ^d^	34.22 ± 3.15 ^c^	55.98 ± 2.57 ^b^	76.33 ± 3.14 ^a^
Triple stain^2^	IPIA	17.76 ± 6.39 ^c^	27.3 ± 5.51 ^b, c^	39.22 ± 4.69 ^a, b^	52.1 ± 4.8 ^a^	75.24 ± 3.83 ^a^
	IPRA	2.72 ± 0.38 ^a^	2.07 ± 0.37 ^a, b^	1.87 ± 0.33 ^a, b, c^	1.38 ± 0.17 ^b, c^	0.83 ± 0.09 ^c^
	DPIA	23.96 ± 4.40	23.50 ± 3.75	23.71 ± 3.41	24.56 ± 3.36	19.89 ± 3.58
	DPRA	57.34 ± 9.03 ^a^	47.14 ± 8.02 ^a, b^	35.22 ± 6.10 ^b, c^	21.97 ± 4.70 ^b, c^	4.04 ± 0.60 ^c^
Fourfold stain^3^	IPIAH	0.64 ± 0.30 ^e^	12.38 ± 0.97 ^d^	23.92 ± 2.27 ^c^	38.38 ± 2.46 ^b^	59.17 ± 2.71 ^a^
	IPIAL	8.59 ± 4.19	8.31 ± 1.54	16.45 ± 5.10	15.15 ± 4.68	12.25 ± 3.00
	IPRAH	0.15 ± 0.08 ^b^	0.23 ± 0.07 ^b^	0.39 ± 0.07 ^a, b^	0.55 ± 0.08 ^a^	0.54 ± 0.08 ^a^
	IPRAL	2.21 ± 0.36 ^a^	1.83 ± 0.34 ^a^	1.21 ± 0.21 ^a, b^	0.83 ± 0.15 ^b^	0.29 ± 0.06 ^b^
	DPIAH	2.29 ± 0.66	2.42 ± 0.51	2.52 ± 0.54	2.96 ± 0.65	1.39 ± 0.40
	DPIAL	19 ± 3.78	18.89 ± 3.52	14.8 ± 3.67	10.94 ± 2.08	8.49 ± 2.2
	DPRAH	2.29 ± 0.66	2.42 ± 0.51	2.52 ± 0.54	2.96 ± 0.65	1.39 ± 0.4
	DPRAL	55.05 ± 9.15 ^a^	44.72 ± 7.90 ^a, b^	31.54 ± 6.25 ^b, c^	18.54 ± 4.30 ^c^	2.65 ± 0.67 ^c^

IP–integrity of plasma membrane, IA–integrity of acrosome, HP–high mitochondrial membrane potential (Δψm), IPIA–simultaneous integrity of plasma and acrosome membranes, IPRA–simultaneous integrity of plasma membrane e reacted acrosome, DPIA–simultaneous damage of plasma membrane and integrity of acrosome, DPRA–simultaneous damage of plasma and acrosome membranes, IPIAH–simultaneous plasma and acrosome membrane integrity and high Δψm, IPIAL—simultaneous plasma and acrosome acrosomal integrity and low Δψm, IPRAH—simultaneous plasma membrane integrity, reacted acrosome and high Δψm, IPRAL—simultaneous plasma membrane integrity, reacted acrosome and low Δψm, DPIAH—simultaneous damaged plasma membrane, acrosome integrity and high Δψm, DPIAL—simultaneous damaged plasma membrane, acrosome integrity and low Δψm, DPRAH—simultaneous damaged plasma membrane, reacted acrosome and high Δψm, DPRAL—simultaneous damaged plasma membrane, reacted acrosome and low Δψm.

^1^Dual stain was performed by the association of Hoechst 33342 with propidium iodide (PI for IP analysis), or *Pisum sativum* agglutinin conjugated to FITC (PSA for IA analysis), or JC-1 fluorescent probes (for HP analysis)

^2^Triple stain was performed by the association of Hoechst 33342 with PI and PSA

^3^Fourfold stain was performed by the association of Hoechst 33342 with PI, PSA and JC-1.

Different letters represent a significant difference (p < 0.05).

### Experiment 2: *In vitro* assay: effect on boar spermatozoa quality of whole sperm-rich fraction seminal plasma addition to frozen-thawed semen

#### Computer-Assisted Sperm Analysis (CASA)

**Fig 4:**
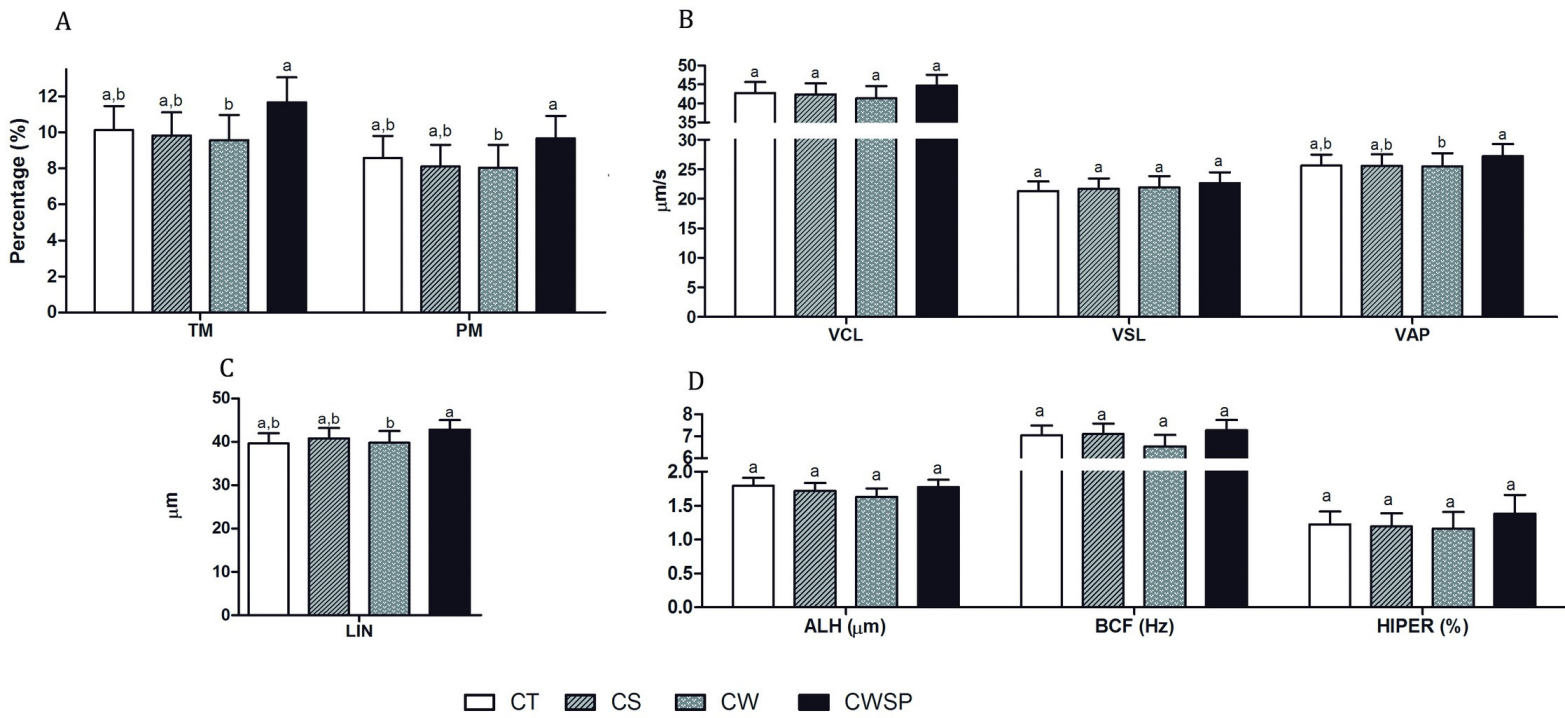
Effect of treatments on computer-assisted sperm analysis (CASA). (A) Total and progressive motility; (B) VCL—curvilinear velocity, VSL—straight-line velocity, VAP—average path velocity; C) LIN—linearity, STR—straightness; (D) ALH—amplitude of lateral head displacement, BCF—beat cross frequency and HIPER—hypermotility. CT—control; CS—centrifuged and suspended in autologous seminal plasma (SP); CW—centrifuged and withdrawn SP; CWSP—CW containing autologous seminal plasma. Different letters represent a significant difference (p < 0.05). CT—control; CS—centrifuged and suspended in autologous seminal plasma (SP); CW—centrifuged and withdrawn SP; CWSP—CW containing autologous seminal plasma. Different letters in the same row represent a difference (p < 0.05) between treatments at the same time.

**Fig 5:**
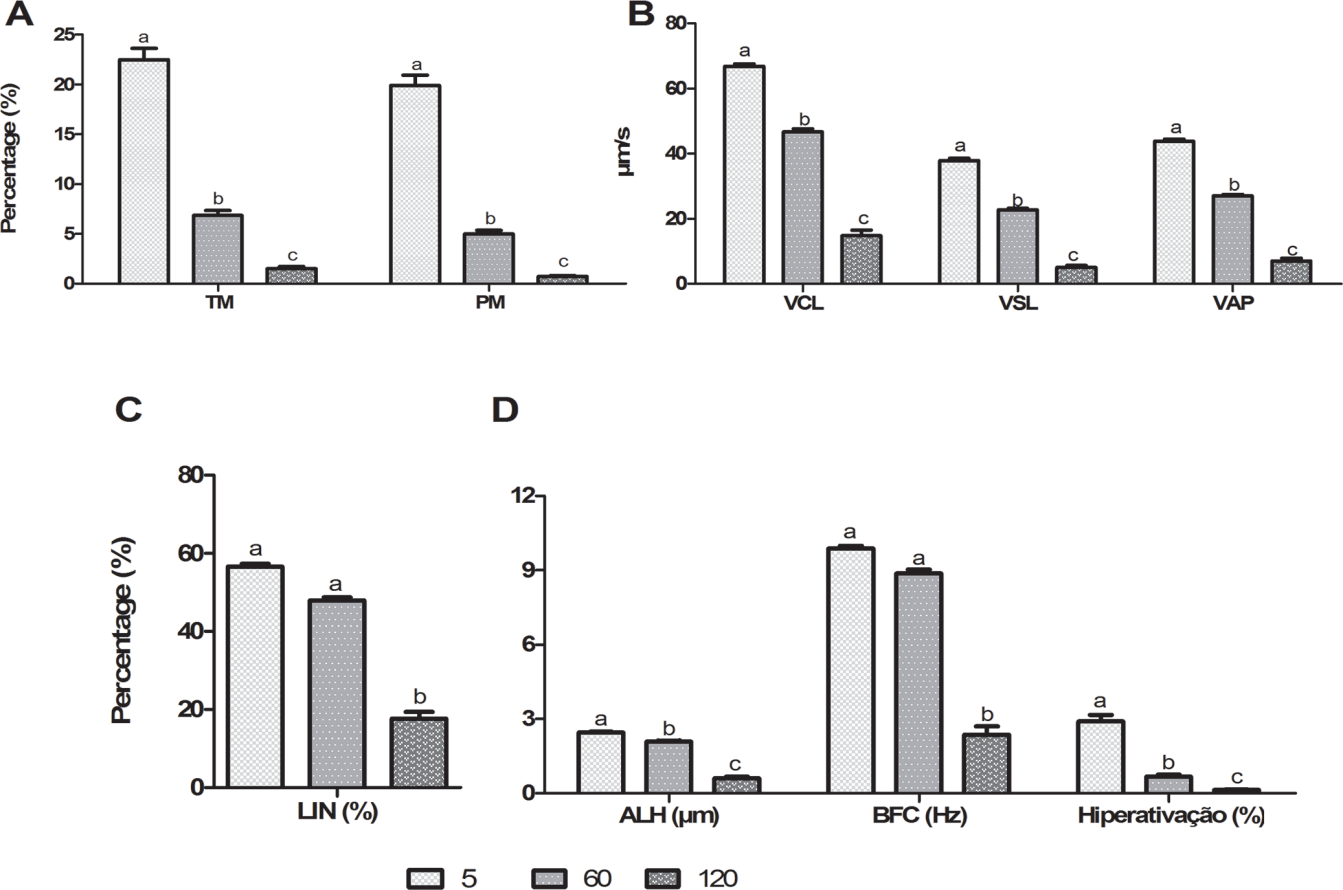
Effect of time on computer-assisted sperm analysis (CASA). (A) Total and progressive motility; (B) VCL—curvilinear velocity, VSL—straight-line velocity, VAP—average path velocity; (C) LIN—linearity, (D) ALH—amplitude of lateral head displacement, BCF—beat cross frequency and HIPER—hypermotility. Different letters represent a significant difference (p < 0.05).

The process of centrifugation pre-freezing did not affect (p > 0.05) any sperm kinematics variables. The addition of SP-SRF at thawed boar semen cryopreserved in absence of SP-SRF improves (p < 0.05) its TM and PM (Fig 4A), VAP (Fig 4B) and LIN (Fig 4C). VCL, VSL (Fig 4B), ALH, BCF and HIPER (Fig 4D) were not influenced (p > 0.05) by any of the treatments. The incubation length affected MT, MP (Fig 5A), VCL, VSL, VAP (Fig 5B), ALH and HIPER (Fig 5D), with 5 minutes being better (p < 0.05) than 60 and 60 better (p < 0.05) than 120. Therefore, no differences (p > 0.05) between 5 and 60 minutes with regard to LIN (Fig 5C) and BCF (Fig 5D) were observed; however, 60 minutes provided better results (p < 0.05) than 120 minutes. Sperm straightness behaved differently over time (p<0.05; interaction time x treatment). In the first five minutes, the addition of seminal plasma or its absence did not alter (p > 0.05) straightness. However, after 120 minutes of incubation, the samples with 10% of SP-SRF maintained a greater straightness when compared with those without seminal plasma (Table 3).

**Table 3:** Mean ± standard error of time vs. treatment interaction for straightness.

	CT	CS	CW	CWSP
5	83.02 ± 0.58^b^	86.31 ± 1.04^a^	87.04 ± 0.7^a^	86.87 ± 0.91^a^
60	82.26 ± 1.11^a^	81.65 ± 1.05^a^	82.56 ± 1.18^a^	81.19 ± 1.08^a^
120	34.73 ± 7.65^a,b^	36.82 ± 7.63^a,b^	25.58 ± 7.5^b^	48.87 ± 6.37^a^

#### Sperm flow cytometry with fourfold stain

**Fig 6:**
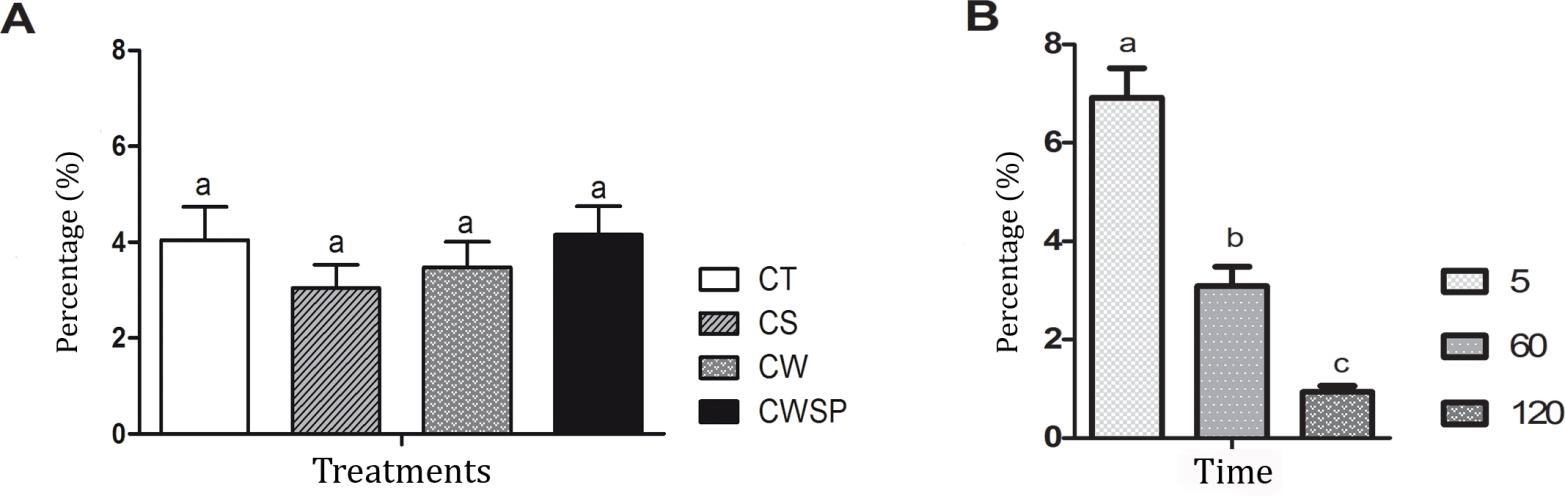
**Effects of treatment (A) and time (B) on plasma and acrosomal membrane integrity and mitochondrial membrane potential.** CT—control; CS—centrifuged and suspended in autologous seminal plasma (SP); CW—centrifuged and withdrawn SP; CWSP—CW containing autologous seminal plasma. IPIAH—plasma and acrosome membrane integrity and high Δψm. Different letters represent a significant difference (p < 0.05).

Membrane integrity evaluated by sperm fourfold stain has no interaction (p > 0.05) between time vs. treatment, thus these effects were separately studied. Pre-freezing centrifugation was not deleterious (p > 0.05) for the sperm populations presenting plasma and acrosomal membrane integrity and high Δψm (IPIAH). This cell category was not improved (p > 0.05) by SP-SRF addition to the thawing medium (Fig 6A), and the absence of SP-SRF was not harmful (p > 0.05; Fig 6A). The proportion of IPIAH sperm cells decreased gradually (p < 0.05) from 5 min to 120 minutes (Fig 6B).

#### Detect of tyrosine phosphorylation on the sperm surface

**Fig 7:**
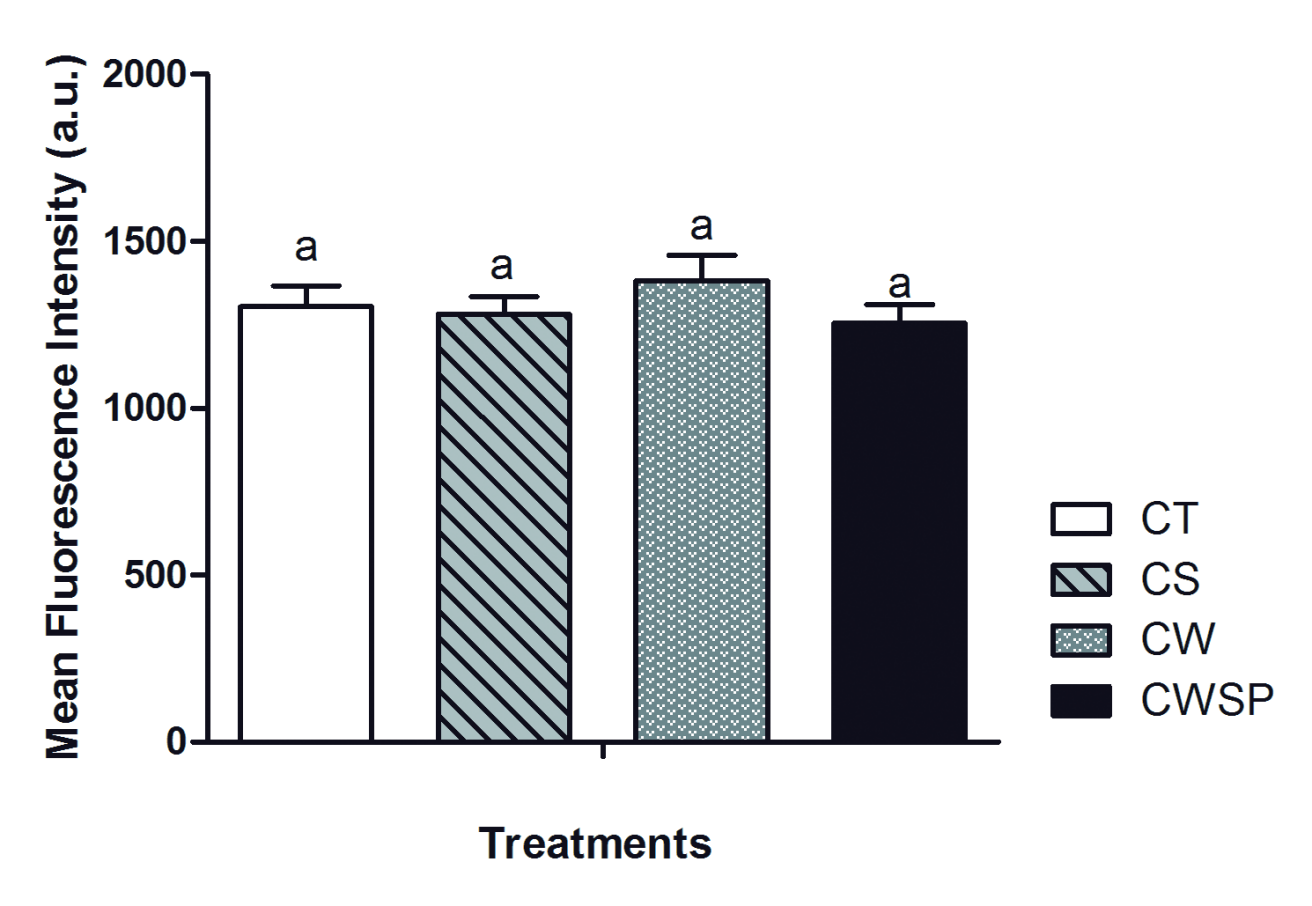
Tyrosine phosphorylation on the surface of spermatozoa. CT—control; CS—centrifuged and suspended in autologous seminal plasma (SP); CW—centrifuged and withdrawn SP; CWSP—CW containing autologous seminal plasma. Different letters represent a significant difference (p < 0.05).

Tyrosine phosphorylation on the sperm surface has no (p > 0.05) interaction between time vs. treatment, thus this effects were separately studied. Tyrosine phosphorylation was not affect (p > 0.05) by pre-freezing centrifugation process. Mean fluorescence intensity of anti-phosphotyrosine antibody conjugated to fluorescein did not decrease due to the addition of SP-SRF to FT boar semen, and it was also unchanged by any of the treatments (p > 0.05 –Fig 7). Live sperm cells showed enhanced (p < 0.05) tyrosine phosphorylation at 5 minutes after thawing than at 60 minutes (5–1408.74 ± 41.86; 60–1193.99 ± 44.56).

### Experiment 3: *In vivo* assay: fertility rate of frozen-thawed boar semen added of whole seminal plasma arising from sperm-rich fraction

Frozen-thawed boar semen fertility (CT—10.59 ± 3.92; CW—9.57 ± 4.92; CWSP—21.29 ± 7.37; p = 0.2225) was not improved by the addition of SP-SRF, and the absence of SP-SRF was not harmful to fertility (p > 0.05). Additionally, embryo recovery was similar between treatments (CT– 74.6 ± 9.76; CW– 76.08 ± 6.57; CWSP– 58.28 ± 9.09; p = 0.1864).

## Discussion

Cryopreservation leads to structural injuries to both sperm plasma and acrosomal membranes as well as functional damage due to capacitation-like changes, which decrease the fertilizing potential of sperm [3]. A single technique for evaluating all of these changes is difficult to develop [14,15]. However, evaluation of additional sperm function parameters in the same sample could estimate the fertilizing potential with greater accuracy than when analyzing individual characteristics [16]. Within this context, this study provides an accurate flow cytometry technique for simultaneously evaluating plasma and acrosomal membrane integrity and mitochondrial membrane potential. When a flow cytometer is available, the sperm fourfold analysis validated in the present work is a great alternative to the simultaneous plasma and acrosomal membrane integrity and mitochondrial membrane potential epifluorescence sperm analysis previously described by De Andrade et al. and Celeghini et al. [17,18]. Both techniques (flow cytometry and epifluorescence) are precise and accurate; however, the flow cytometry technique has the advantage of being an objective analysis, and a large number of cells can be counted in a short period [21]. Nonetheless, compared to the flow cytometry technique, epifluorescence is less expensive. Therefore, as both simultaneous sperm plasma and acrosomal membrane integrity and mitochondrial membrane potential analyses can be performed with great accuracy, the choice should be based on the laboratory conditions.

Furthermore, the simultaneous assessment of mitochondrial potential, plasma and acrosome membrane integrity by sperm fourfold stain is a great alternative to dual or triple stain evaluation of sperm membranes function. In general, the standard sperm analysis is performed as a dual stain in which a single sperm compartment was evaluated or triple stain that allows the evaluation of two sperm compartments (plasma and acrosome membranes) simultaneously [13,27]. However, evaluation of additional sperm function parameters in the same sample (as sperm fourfold stain) restricts the target population, minimizing overrate of the single or dual sperm compartments assay. Sperm evaluation by fourfold stain technique is able to minimize overrates of potentially fertile spermatozoa compared with triple stain; since the first is able to restrict the potentially fertile spermatozoa, showing a population with 16% less of cell integrity than the second. It is because fourfold stain is able to detect a sperm population that goes undetected by triple stain. Those 16% of cells in triple stain matches the sperm population that has plasma and acrossomal membranes integrity but low mitochondrial membrane potential, which are unable to fertilize the oocyte. As far as know the maintenance of integrity of mitochondrial membrane potential, plasma and acrosome membranes are essential to allow the sperm meeting and fertilizing a mature oocyte [41].

Although SP withdrawal results in changes in sperm kinetics [42,43], this is an essential step to increase sperm concentration [44] to allow the use of 0.25 and 0.5 mL straws for storage. Nevertheless, the addition of SP-SRF at thawing can improve the total and progressive motility of samples frozen in absence of SP-SRF. Post-thawed addition, as well as, cryopreservation with SP-SRF (control samples) had no differences in motility characteristics, leading us to believe that the effect of SP-SRF is independent of the time of addition (before or after cryopreservation). This disagrees with the notion that maintaining SP during cryopreservation is deleterious to sperm quality [10]. Although our study was not able to show a decrease in hyperactive sperm due to SP-SRF, kinematic characteristics were improved, as revealed by more linear movement, improving the average path velocity and linearity compared to samples without SP-SRF.

Semen cryopreservation drastically damages boar sperm cells [45], and alternatives for improving this technique have been widely investigated. Seminal plasma emerged as a possible solution many years ago. This non-cellular semen fraction decreases the harmful effects of cryopreservation due to its composition [33]. Sperm-rich fraction and sperm-peak portion have advantages compared to bulk ejaculate, such as better resistance to cold shock, premature acrosome reactions, and a more stable plasma membrane [46]. These specific fractions have a relatively low level of proteins from accessory sex glands, high levels of epididymis fluid and a low level of bicarbonate [47]. However, improvement due to SP-SRF was not observed in our study with regard to potentially fertile spermatozoa: sperm populations with simultaneous plasma and acrosomal membrane integrity and high mitochondrial membrane potential; which is contrary to what has been previously described in boars [10–12,48] and also described in stallions [49]. However, those experiments evaluated sperm membranes integrity by dual or triple stain. As demonstrated in the present experiment those techniques could overrate the potentially fertile spermatozoa. Our experiment demonstrated that the addition of SP-SRF at FT boar semen on the sperm population was not able to increase the real fertile potential assessed by sperm fourfold stain.

Increased tyrosine phosphorylation appears to be a capacitation cascade event [50] that is correlated with the presence of calcium and bicarbonate [51]. Despite mounting evidence that seminal plasma has an important role in spermatic cell physiology [52], our results did not show a beneficial effect of SP-SRF with respect to tyrosine phosphorylation, contrary to what is described in stallions [32]. Furthermore, boar SP-SRF has a low bicarbonate concentration [47], a factor that can explain the low level of tyrosine phosphorylation in our samples. Tyrosine phosphorylation increases have been correlated with sperm hyperactivation [36], but neither of these sperm characteristics was affected by the addition of SP-SRF.

Seminal plasma removal is a widespread procedure in state-of-the-art boar semen cryopreservation protocols; it is essential to concentrate samples [43]. However, pre-freezing centrifugation affects the post-thawed semen quality [53]. In this way, the effect of absence of seminal plasma at thawing, compared to samples that had seminal plasma added at thawing, could be misunderstood if the results were not carefully interpreted. The inclusion of CS treatment in the experimental design allows the verification of centrifugal force effect on sperm characteristics. The absence of centrifugation pre-freezing deleterious effect to boar spermatozoa characteristics allowed us to drawn the conclusion that the effects of seminal plasma absence, compared to samples that had seminal plasma added at thawing, is caused byseminal plasma per se, without any effect of centrifugation.

Some aspects of sperm physiology have been highly correlated with seminal plasma proteins. Members of the spermadhensin superfamily, the most abundant (90%) proteins in boar seminal plasma [53,54], have been associated with oviduct reservoir formation [55] via the binding of AQN-1 to mannose residues of the apical membrane fractions of the oviduct epithelium [56]. During capacitation, AQN and AWN spermadhensins function as decapacitation factors [57] by acting as cholesterol acceptors, thereby increasing plasma membrane fluidity. Finally, spermadhensins act in gamete interaction [58], possibly through AQN-3 and AWN recognition and binding [59–61]. This leads us to believe that the addition of SP-SRF to FT boar semen could increase fertility, as described by Hernández et al., Garcia et al., and Okazaki et al., [12,62,63]. However, this work was not able to statistically demonstrate this beneficial effect. Moreover, as demonstrated bellow, the addition of SP-SRF at FT boar semen was not effective to improve the potentially fertile spermatozoa, which could explain the absence in fertility improvement.

In conclusion, (1) the simultaneous flow cytometry assay of plasma and acrosomal membrane integrity and mitochondrial membrane potential can be achieved with Hoechst 33342, propidium iodide, PSA-FITC and JC-1 staining. (2) Addition of SP-SRF on FT boar semen improves motility characteristics of boar semen cryopreserved in absence of SP-SRF, without effect on plasma and acrossomal membrane integrity, mitochondrial potential as well as tyrosine phosphorylation. Absence of SP-SRF is not harmful to integrity of acrosomal and plasma membranes, neither for mitochondrial potential. Moreover, absence of SP-SRF did not alter tyrosine phosphorylation on sperm surface. Although SP-SRF absence was not able to reduce fertility rates, females inseminated with samples containing SP-SRF had 2.2 more fertilized structures than those inseminated with samples not containing SP-SRF. (3) Beyond that, centrifugation has not effect on the post-thawed boar semen quality, enabling separation of the centrifugation and the SP-SRF effects. Additional studies of whole sperm-rich seminal plasma addition are needed to clarify these effects.

## Supporting Information

S1 Fig Fluorochrome controls for manual flow cytometry compensation. (A) Sample subjected to three cycles of flash freezing (FF) in liquid nitrogen and then slowly thawed to induce damage to the plasma membranes followed by propidium iodide staining (PI positive). (I) Histogram showing the absence of PSA-FITC fluorescence; (II) histogram showing the absence of JC-1 orange fluorescence; (III) histogram showing the IP (plasma membrane integrity, PI negative) and DP (damaged plasma membrane, PI positive) gates and the concentration of the sperm population on the DP gate; (IV) double dot plot for evaluating the mitochondrial membrane potential (Δψm—y axis) and acrosome integrity (x axis); the green dot plot originated from the IP gate, and the red dot plot originated from the DP gate; the populations represented in the dot plots match the sperm characteristics shown in previous histograms. (B) Sample subjected to FF, as described above, to induce acrosomal damage and stained with *Pisum sativum* agglutinin conjugated to FITC (PSA-FITC positive). (I) Histogram showing positive PSA-FITC fluorescence; (II) histogram showing the absence of JC-1 orange fluorescence; (III) histogram showing the IP and DP gates and the concentration of the sperm population on the IP gate; (IV) the populations represented in the dot plots match the sperm characteristics shown in previous histograms. (C) Viable sample with high Δψm stained with JC-1 (orange fluorescence of J-aggregates). (I) Histogram showing the absence PSA-FITC fluorescence; (II) histogram showing JC-1 orange fluorescence; (III) histogram showing the IP and DP gates and the concentration of sperm population on the IP gate; (IV) the populations represented in the dot plots match the sperm characteristics shown in previous histograms. (D) Viable sample simultaneously stained with PI and PSA-FITC to separate the green fluorescence from PSA-FITC and the red fluorescence from PI. (I) Histogram showing negative PSA-FITC fluorescence; (II) histogram showing the absence of JC-1 orange fluorescence; (III) histogram showing the sperm population on the IP and DP gates; (IV) the populations represented in the dot plots match the sperm characteristics shown in previous histograms. (E) Viable samples simultaneously stained with PI and JC-1 to separate the red fluorescence from PI and the orange fluorescence from JC-1 (J-aggregates). (I) Histogram showing the absence of PSA-FITC fluorescence; (II) histogram showing JC-1 orange fluorescence; (III) histogram showing the IP and DP gates and the concentration of the sperm population on the IP gate; (IV) the populations represented in the dot plots match the sperm characteristics shown in previous histograms. (F) Samples subjected to FF, as described above, with low Δψm and reacted acrosomal membrane stained with PSA-FITC and JC-1 to separate the green fluorescence from PSA-FITC and the orange (J-aggregates) and green (J-monomers) fluorescence from JC-1. I) Histogram showing positive PSA-FITC fluorescence; (II) histogram showing the absence of JC-1 orange fluorescence; (III) histogram showing the IP and DP gates; (IV) the populations represented in the dot plots match the sperm characteristics shown in previous histograms. IPIAH: plasma and acrosomal membrane integrity and high Δψm; IPIAL: plasma and acrosome acrosomal integrity and low Δψm; IPRAH: plasma membrane integrity, reacted acrosome and high Δψm; IPRAL: plasma membrane integrity, reacted acrosome and low Δψm; DPIAH: damaged plasma membrane, acrosome integrity and high Δψm; DPIAL: damaged plasma membrane, acrosome integrity and low Δψm; DPRAH: damaged plasma membrane, reacted acrosome and high Δψm; DPRAL: damaged plasma membrane, reacted acrosome and low Δψm.
(TIF)

S1 Dataset Minimal dataset underlying the findings. CT—control; CS—centrifuged and suspended in autologous seminal plasma (SP); CW—centrifuged and withdrawn SP; CWSP—CW containing autologous seminal plasma.
(ZIP)
