# Supplementary figures and images for: Novel Flow Cytometry Analyses of Boar Sperm Viability: Can the Addition of Whole Sperm-Rich Fraction Seminal Plasma to Frozen-Thawed Boar Sperm Affect It?
Source: PLoS One. 2016 Aug 16;11(8):e0160988. doi: 10.1371/journal.pone.0160988 (PMC4987046; doi:10.1371/journal.pone.0160988)

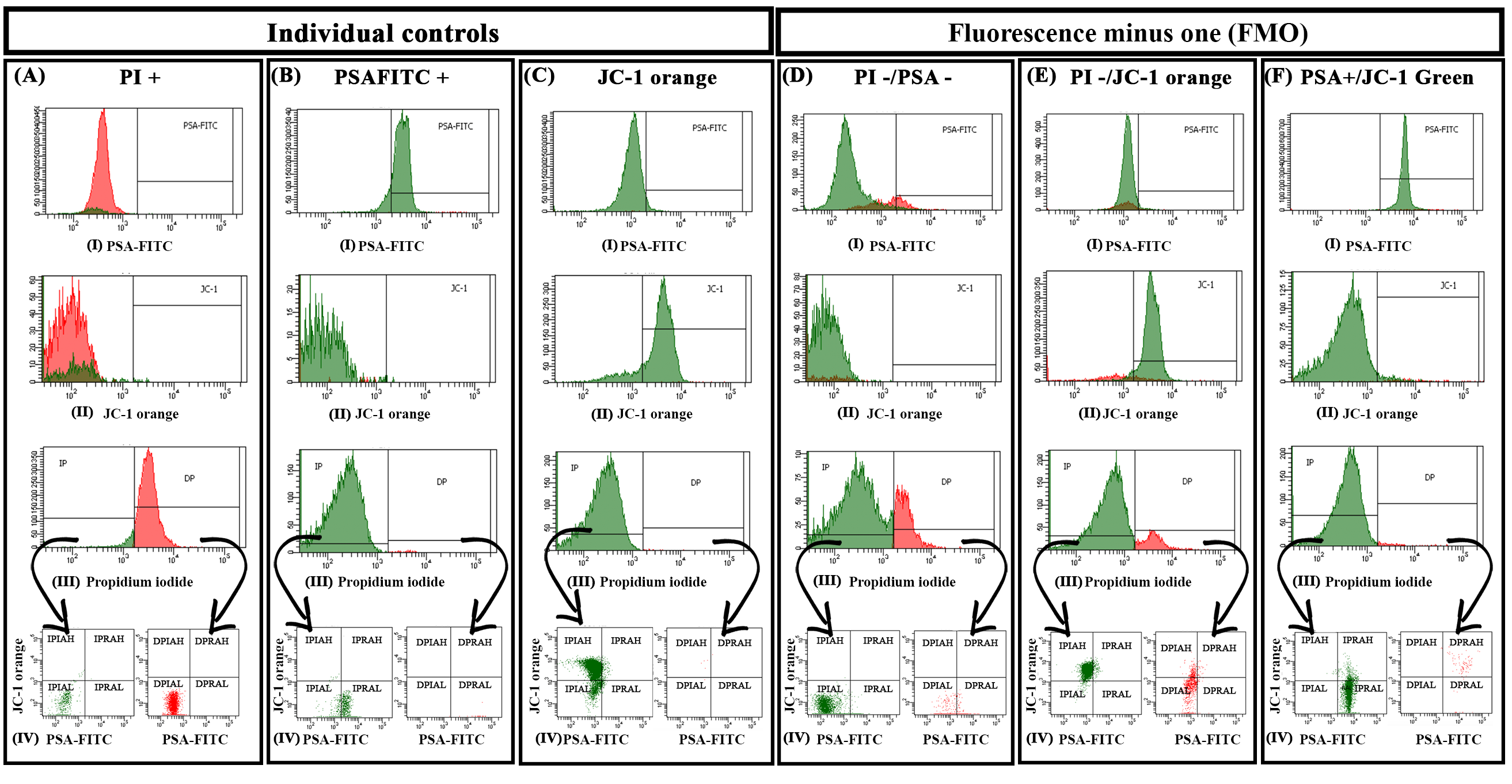

Supplement: S1 Fig — (A) Sample subjected to three cycles of flash freezing (FF) in liquid nitrogen and then slowly thawed to induce damage to the plasma membranes followed by propidium iodide staining (PI positive). (I) Histogram showing the absence of PSA-FITC fluorescence; (II) histogram showing the absence of JC-1 orange fluorescence; (III) histogram showing the IP (plasma membrane integrity, PI negative) and DP (damaged plasma membrane, PI positive) gates and the concentration of the sperm population on the DP gate; (IV) double dot plot for evaluating the mitochondrial membrane potential (Δψm—y axis) and acrosome integrity (x axis); the green dot plot originated from the IP gate, and the red dot plot originated from the DP gate; the populations represented in the dot plots match the sperm characteristics shown in previous histograms. (B) Sample subjected to FF, as described above, to induce acrosomal damage and stained with Pisum sativum agglutinin conjugated to FITC (PSA-FITC positive). (I) Histogram showing positive PSA-FITC fluorescence; (II) histogram showing the absence of JC-1 orange fluorescence; (III) histogram showing the IP and DP gates and the concentration of the sperm population on the IP gate; (IV) the populations represented in the dot plots match the sperm characteristics shown in previous histograms. (C) Viable sample with high Δψm stained with JC-1 (orange fluorescence of J-aggregates). (I) Histogram showing the absence PSA-FITC fluorescence; (II) histogram showing JC-1 orange fluorescence; (III) histogram showing the IP and DP gates and the concentration of sperm population on the IP gate; (IV) the populations represented in the dot plots match the sperm characteristics shown in previous histograms. (D) Viable sample simultaneously stained with PI and PSA-FITC to separate the green fluorescence from PSA-FITC and the red fluorescence from PI. (I) Histogram showing negative PSA-FITC fluorescence; (II) histogram showing the absence of JC-1 orange fluorescence; (III) [file pone.0160988.s001.tif]
